# Genomic Marks Associated with Chromatin Compartments in the CTCF, RNAPII Loop and Genomic Windows

**DOI:** 10.3390/ijms222111591

**Published:** 2021-10-27

**Authors:** Teresa Szczepińska, Ayatullah Faruk Mollah, Dariusz Plewczynski

**Affiliations:** 1 Centre of New Technologies, University of Warsaw, Banacha 2c, 02-097 Warsaw, Poland; t.szczepinska@cezamat.eu (T.S.); afmollah@aliah.ac.in (A.F.M.); 2 CEZAMAT, Warsaw University of Technology, Poleczki 19, 02-822 Warsaw, Poland; 3 Department of Computer Science and Engineering, Aliah University, Kolkata 700160, India; 4 Faculty of Mathematics and Information Science, Warsaw University of Technology, Koszykowa 75, 00-662 Warsaw, Poland

**Keywords:** 3D genome structure, chromatin compartments, epigenetic modifications, open chromatin, H3K4me1, H3K79me2, H3K9me3, H4K20me1, H3K27me3, GC content

## Abstract

The nature of genome organization into two basic structural compartments is as yet undiscovered. However, it has been indicated to be a mechanism of gene expression regulation. Using the classification approach, we ranked genomic marks that hint at compartmentalization. We considered a broad range of marks, including GC content, histone modifications, DNA binding proteins, open chromatin, transcription and genome regulatory segmentation in GM12878 cells. Genomic marks were defined over CTCF or RNAPII loops, which are basic elements of genome 3D structure, and over 100 kb genomic windows. Experiments were carried out to empirically assess the whole set of features, as well as the individual features in classification of loops/windows, into compartment A or B. Using Monte Carlo Feature Selection and Analysis of Variance, we constructed a ranking of feature importance for classification. The best simple indicator of compartmentalization is DNase-seq open chromatin measurement for CTCF loops, H3K4me1 for RNAPII loops and H3K79me2 for genomic windows. Among DNA binding proteins, this is RUNX3 transcription factor for loops and RNAPII for genomic windows. Chromatin state prediction methods that indicate active elements like promoters, enhancers or heterochromatin enhance the prediction of loop segregation into compartments. However, H3K9me3, H4K20me1, H3K27me3 histone modifications and GC content poorly indicate compartments.

## 1. Introduction

The genetic information of eukaryotes is stored in a cell nucleus in the form of a nucleoprotein complex of DNA and histones, known as chromatin. A basic aspect of chromatin structure is that each chromosome occupies a discrete volume, forming a “chromosome territory” [1]. Proximity-based ligation techniques coupled with massively parallel sequencing (Hi-C) have provided evidence for topologically associating domains (TADs). A TAD is defined as a region of a chromosome that shares many interactions within it, but significantly fewer interactions with the adjacent and other more distal TADs [2,3]. The concept of TADs that are of a size ~ 1 Mbp is in concordance with microscopic evidence [4]. The Hi-C map of the human genome at a higher, kilo-base resolution reveals the inner structure of TADs [5]. The observed domains ranged in size from 40 kb to 3 Mb (median size 185 kb). Many of them are loops mediated by CCCTC-binding factor (CTCF). In different experiments on the same cell line (GM12878), chromatin interaction analysis by paired-end tag sequencing (ChIA-PET) strategy allowed the comprehensive mapping of higher-order chromosome folding and specific chromatin interactions mediated by CCCTC-binding factor (CTCF) and RNA polymerase II (RNAPII) [6]. CTCF-mediated chromatin contact domains retrieved from ChIA-PET were highly concordant with TADs detected by Hi-C method [6].

Rao et al. showed that domains fold in a way that means they form larger subcompartments with at least six distinct patterns of histone marks. These compartments can be assigned to two general compartments A and B (identified before from 1 Mb resolution Hi-C matrices [7]. Switching was observed between subcompartments roughly every 300 kb and between the A and the B compartment roughly every 400 kb [7]. Both subcompartments in compartment A were shown to be gene dense, have highly expressed genes, harbor activating chromatin marks such as H3K36me3, H3K79me2, H3K27ac and H3K4me1 and to be depleted at the nuclear envelope and at nucleolus associated domains (NADs). Four subcompartments of compartment B were found. Subcompartment B1 correlates positively with H3K27me3 and negatively with H3K36me3 and was suggested to be facultative heterochromatin. Subcompartment B2 includes 62% of pericentromeric heterochromatin and is enriched at the nuclear lamina and at NADs. Subcompartment B3 tends to lack all of the above-noted marks, suggesting ordinary heterochromatin. It is enriched at the nuclear lamina, but strongly depleted at NADs. Subcompartment B4 spans only 0.3% of the genome, only on chromosome 19, and has strong enrichment for both activating chromatin marks, such as H3K36me3, and heterochromatin-associated marks, such as H3K9me3 and H4K20me3 [5]. So far, many mechanisms for the formation of compartments have been proposed, such as attraction of heterochromatin to the nuclear lamina, preferential attraction of similar chromatin to each other, higher levels of chromatin mobility in active chromatin and transcription-related clustering of euchromatin (reviewed in [8]). However, these hypotheses have remained inconclusive.

**Figure 1:**
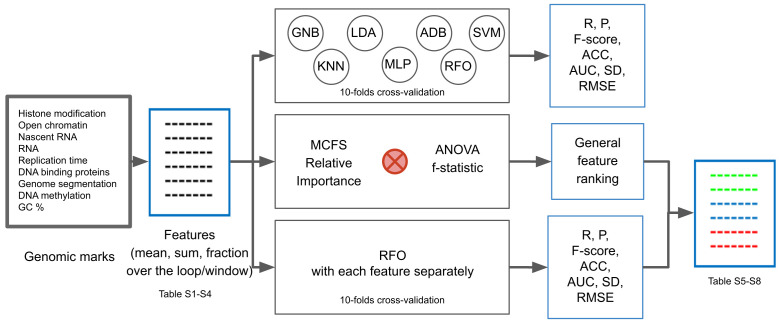
Feature evaluation diagram.

In this paper, we analyse the association of chromatin marks with compartments that have the additional details. We identify the non-redundant genomic marks optimal for classification of CTCF and RNAPII loops as well as genomic windows into compartment A or compartment B based on a consensus of the relative importance of Monte Carlo feature selection method (MCFS) and analysis of variance (ANOVA) f-statistic feature fitness measure (Figure 1). MCFS was originally verified on a classification of cancer patients based on gene expression microarray data [9]. In that task, there were a few thousand features in the classification of a few dozen samples. Our task had a much more informative training set; we made a selection from a few hundred features based on a thousand samples. Similar to that task, a classifier per se was not crucial here; rather, selection of informative features was the most important issue. So far, only correlation was used to describe the relation between genomic features and compartmentalization. We show the relative importance of the features. The main motivation of this analysis was to better understand the essence of the phenomenon of genome compartmentalization.

## 2. Results

We collected a range of genomic data that characterize chromatin state and chromatin binding proteins in this cell line. These are mostly ChIP-seq data of histone modifications (H3K27ac, H3K27me3, H3K36me3, H3K4me1, H3K4me2, H3K4me3, H3K79me2, H3K9ac, H3K9me3, H4K20me1) and transcription factors from the ENCODE project [10]. Additionally, we collected data about DNA methylation, open chromatin state (DNase-seq, FAIRE-seq and ATAC-seq), RNAs (RNA-seq), and nascent RNAs (GRO-seq, Bru-seq). Moreover, we used ENCODE combined data: open chromatin synthesis and genome regulatory segmentation (ChromHMM, Segway methods and a compilation of both). We also collected the GC percentage information. Each loop has one number assigned for each feature. It was either a fraction of the loop covered with the peak signal, the mean or the sum of the peak signal along the whole loop or the mean of the whole signal along the loop without peak identification. Therefore, one genomic mark was represented by several features. Altogether, we created 428 features from 142 experiments (Supplementary Information). We used compartment genomic coordinates discovered in Hi-C high-resolution (1 kb) experiments [5].

Firstly, we empirically assessed the whole set of genomic marks to check how good they are at characterizing genomic compartment A and B. To do so, instead of relying on a single classification model, we applied a range of classifiers such as Gaussian Naïve Bayes (GNB), K-Nearest Neighbors (KNN), Linear Discriminant Analysis (LDA), AdaBoost (ADB), Support Vector Machine (SVM), Multi-layer Perceptron (MLP) and Random Forest (RFO), and followed a cross-validation strategy. To report the performance of the above classification models, several standard evaluation metrics such as Recall (R), Precision (P), harmonic mean of R and P, i.e., F-Score, Accuracy (ACC), Area Under Receiver Operating Characteristic Curve (AUC for short), Standard Deviation (SD) of AUCs across different folds of cross-validation, and Root Mean Square Error (RMSE) were employed. In the ideal scenario, measures like R, P, F-Score, ACC and AUC should be 1.0, and measures like SD and RMSE should be 0.0.

Then, we identified genomic marks important for classification of compartments. We used the relative importance (RI) of the feature values, an important statistical measure from the Monte Carlo Feature Selection method and ANOVA test, to indicate the most informative features for a classifier [9,11]. The ranking of the features was based on the product of RI and ANOVA f-statistic. Along with that, individual feature performance in classifying compartments was evaluated with the RFO classifier, as it was found to be the best performer in most of our experiments in terms of AUC. It may be noted that in all the classification experiments, the 10-fold cross-validation strategy was followed to ensure robust and consistent findings. This was conducted across two types of CTCF loops, with CTCF motif in convergent and tandem orientation, RNAPII loops and 100 kb genomic windows separately (Tables S1–S4). Finally, we analysed the findings and examined the consistency of the identified genomic marks.

### 2.1. CTCF Convergent Loops

**Figure 2:**
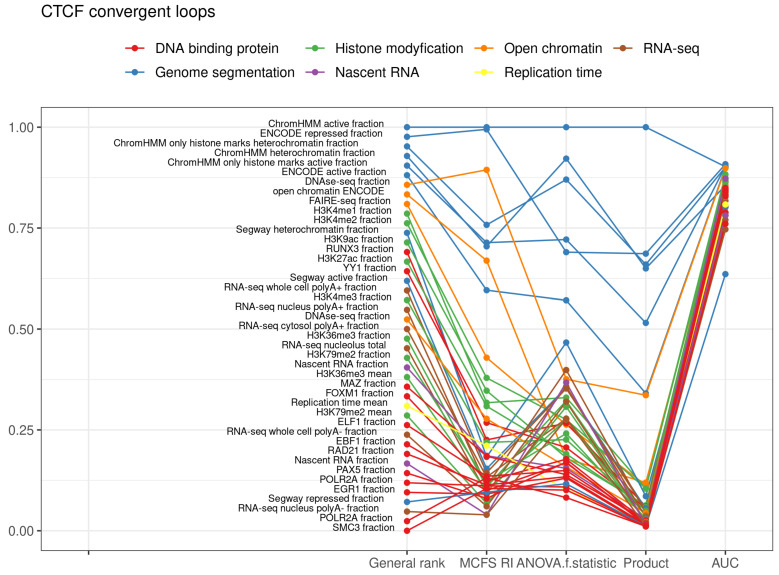
Top decile of the most important features for classification of CTCF convergent loops into compartments.

Initially, compartmentalization was examined on the basis of all the genomic marks of the CTCF convergent loops with a wide range of classification models. We obtained average values of 0.9008 (recall), 0.9076 (precision), 0.9008 (f-score), 0.9008 (accuracy), 0.9575 (AUC), 0.0195 (standard deviation of AUCs for 10-fold) and 0.3102 (RMSE), as shown in Table 1. This reflects that the genomic marks are strong indicators for compartmentalization. Subsequently, the importance of genomic marks, and features, as their representation, was evaluated (Figure 2, Table S5). At the top of the ranking, there were features that are combinations of simple features like fraction of active, repressed, or heterochromatin, which came before simple features like DNase-seq or FAIRE-seq fraction (Table 2). Simple features are those that are a single experiment measurement, e.g., measurement of the level of histone methylation of one type or open chromatin. Features that are combinations are the results of the processing of measurements from many experiments, e.g., ChromHMM method [12]. This method annotates promoters, enhancers, heterochromatin, etc., on the basis of histone modifications and open chromatin measurements. Such chromatin state prediction methods enhance the prediction of the segregation of the loops into compartments. The top ranked simple feature is DNase-seq measurement of open chromatin. The top-ranked histone modification is histone H3 lysine 4 monomethylation (H3K4me1) (Table S5). A similar level of prediction gives the classification based only on dimethylation of this lysine (H3K4me2), H3 lysine 9 acetylation (H3K9ac), H3 lysine 27 acetylation (H3K27ac) and H3 lysine 4 trimethylation (H3K4me3). All these modifications are known to be correlated with active chromatin state [13,14]. These genomic marks performed the best, being represented by the fraction of the loop length that is covered by the ChIP-seq signal. Histone modifications that poorly indicated compartments were H3K9me3, H4K20me1, H3K27me3 (positions 336, 119 and 104 in ranking, respectively). These histone modifications are often associated with heterochromatin [15,16]. It is important to mention that GC content, which was good for indicating which of two compartments is active, was not good for the prediction of compartment association for a particular region (rank 409, AUC = 0.511 SD = 0.0146). Among the proteins identified by ChIP-seq, the best for compartment prediction were RUNX3 and YY1 proteins (14 and 16 in rank). RUNX3 is a transcription factor found in several enhancers and promoters, and can either activate or suppress transcription, as reviewed in [17,18]. YY1 contributes to enhancer-promoter structural interactions in a manner analogous to DNA interactions mediated by CTCF [19]. All proteins with AUC > 0.8) are RUNX3, YY1, MAZ, FOXM1, ELF1, EBF1, PAX5, POLR2A, EGR1, MXI1, POU2F2, ZNF384, WRNIP1, TCF12, CREB1, PML, CHD2, SPI1, SP1, TAF1 (in order of general ranking).

### 2.2. CTCF Tandem Loops

**Figure 3:**
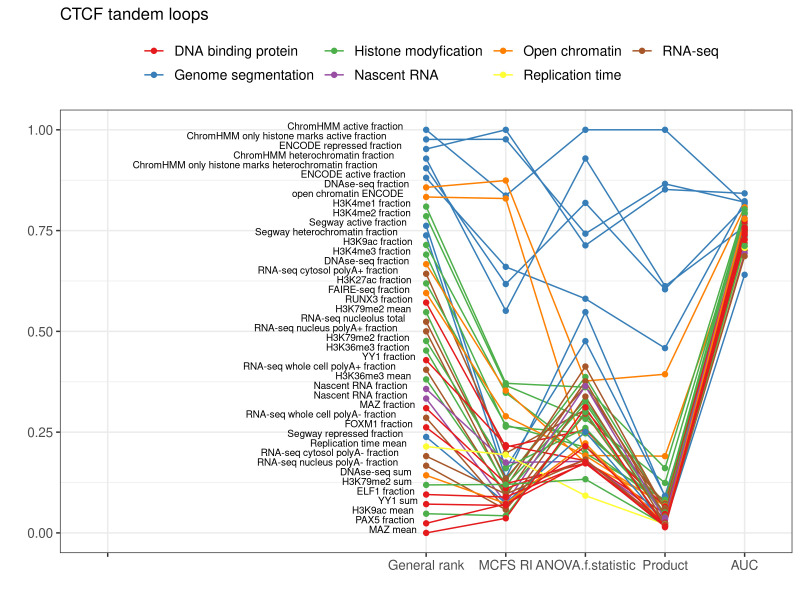
Top decile of the most important features for classification of CTCF tandem loops into compartments.

There were fewer tandem CTCF loops than convergent loops (33.1% vs. 64.5%) and a greater percentage of them were in compartment A (72.9% vs. 62.7%). As reflected in Table 3, the same genomic marks can distinguish tandem loops into Compartment A or B, like the convergent loops. Average values of 0.8543 (recall), 0.8805 (precision), 0.8653 (f-score), 0.8643 (accuracy), 0.9208 (AUC), 0.0269 (standard deviation of AUCs for 10-fold) and 0.3531 (RMSE) were obtained. This signifies that the classification power of these genomic marks is marginally lower in the case of tandem loops in comparison to convergent loops. However, the ranking of features for CTCF tandem loops is very similar to that for CTCF convergent loops, which suggests their similar role in compartmentalization (Figure 3, Table S6).

### 2.3. RNAPII Loops

**Figure 4:**
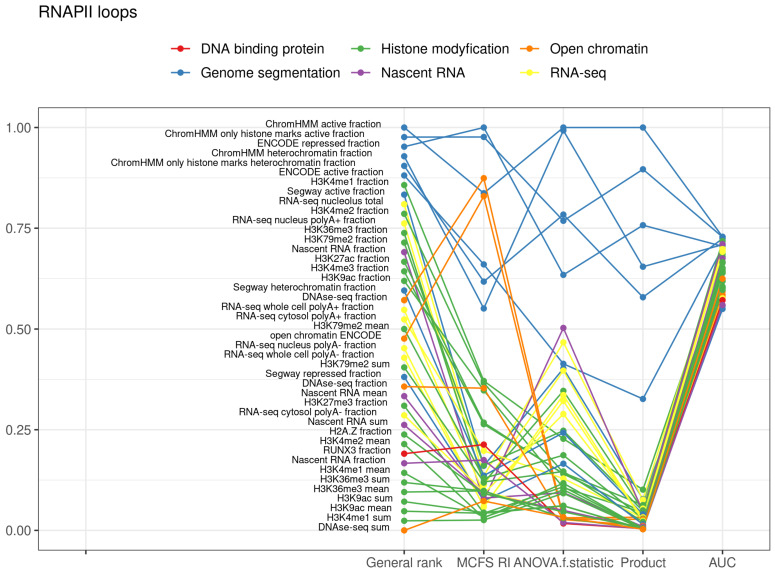
Top decile of most important features for classification of RNAPII loops into compartments.

Compartment A was more active in transcription, but still 19.6% of RNAPII loops were in compartment B. Based on all the features for RNAPII loops, the classifiers yielded average values of 0.8179 (recall), 0.8486 (precision), 0.8091 (f-score), 0.8179 (accuracy), 0.8374 (AUC), 0.0597 (standard deviation of AUCs for 10-fold) and 0.3950 (RMSE) (Table 4), reflecting differences in the features in RNAPII loops in compartment A and B. Similar to CTCF convergent and tandem loops at the top of the feature ranking, there were features that were combinations of simple features describing active, repressed or heterochromatin regions (Figure 4, Table S7). Histone modifications possessed similar importance for classification to their importance in classification of CTCF loops. However, there was a higher importance of H3K27me3, H3K79me2, and H4K20me1 (Table 2 and Table S7). Moreover, the RNA-seq experiment (position 9 in the ranking) and nascent RNA measurements by Bru-seq experiment (position 14 in the ranking) were more important for classification. In contrast, predictions by open chromatin were less powerful. The prediction of compartments for RNAPII loops based on GC content was only slightly better than for CTCF loop (AUC 0.591 SD 0.0162 vs. AUC 0.511 SD 0.0146 and AUC 0.502 SD 0.0235 for CTCF convergent and tandem loops, respectively).

### 2.4. Genomic Windows

**Figure 5:**
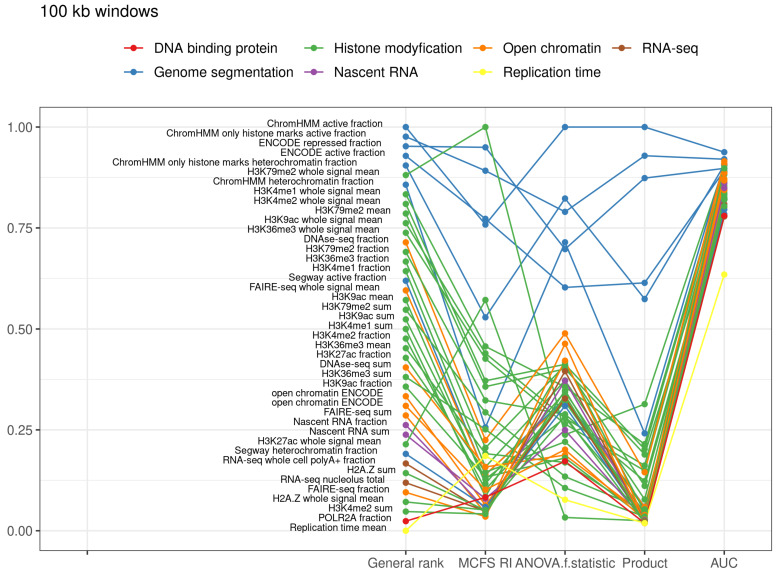
Top decile of the most important features for classification of 100 kb windows into compartments.

For comparison of the classification of loops into compartments, we also performed classification of the 100 kb genomic windows covering the whole genome. The classifiers based on all the features also performed very well, similar to the case of CTCF convergent loops, with average values of 0.8851 (recall), 0.8885 (precision), 0.8844 (f-score), 0.8851 (accuracy), 0.9514 (AUC), 0.0127 (standard deviation of AUCs for 10-fold) and 0.3315 (RMSE) (Table 5). Differently from the classification of genomic windows compared with the classification of loops, there was a very high position in the ranking of histone 3 lysine 79 dimethylation (H3K79me2) (Figure 5, Table 2 and Table S8). Methylation of H3K79 is involved in the regulation of telomeric silencing, cellular development, cell-cycle checkpoint, DNA repair, and regulation of transcription [20].

## 3. Discussion

The chromatin modification H3K4me1 is a hallmark of the initial stage of enhancer activation, while the appearance of active chromatin marks such as H3K27ac defines an active state [21]. For example, H3K4me1 is used to distinguish active from poised enhancers when combined with H3K27ac and H3K27me3, respectively. These additional hallmarks are much less important for the prediction of compartmentalization than H3K4me1. H3K4me2 reliably defines the transcription factor binding regions [22]. It is enriched in promoters and some enhancers [23,24]. H3K9ac is enriched in actively transcribed promoters and active enhancers [25]. Therefore, histone modifications associated with active chromatin are very good predictors of compartmentalization, together with open chromatin measured by DNase-seq. In contrast, chromatin modification associated with heterochromatin (condensed chromatin) like H3K9me3, H3K27me3, H4K20me1 [26] is not useful in compartment prediction. This suggests the broader range of functions associated with those genomic marks that are important in both compartments. Interestingly, heterochromatin state or Polycomb repressed state give very good compartment predictions. The method of prediction of those states is based on a multivariate Hidden Markov Model (HMM) that explicitly models mark combinations [12]. This suggests that combinations of histone modifications, associated with condensed and active chromatin, and not single chromatin marks, are necessary to uniquely describe compartment B. Interactions between heterochromatin were recently shown to play the central role in establishing compartmentalization by phase separation [8], which is in agreement with our study. However, among candidates for mediators of heterochromatin–heterochromatin interactions, the authors suggested modified histones, which is not supported by our analysis.

## 4. Materials and Methods

All the data are for GM12878 cell line and for hg19 human genome assembly. GM12878 is a lymphoblastoid cell line produced from the blood of a female donor with northern and western European ancestry by EBV transformation [27]. It is one of the Tier 1 ENCODE cell lines, so the vast amount of sequencing data, including transcriptome, chromatin immunoprecipitation-sequencing for histone marks, and transcription factors are available for this line together with genome regulatory segmentation. This cell line has a relatively normal karyotype and it represents the mesoderm cell lineage.

**Compartment association.** We used compartment genomic coordinates discovered in Hi-C high-resolution (1 kb) experiments where subcompartments were associated with 100 kb windows [5]. Subcompartment A1 and A2 were associated with compartment A and compartment B1, B2, B3, B4 were associated with compartment B.

**CTCF and RNAPII loops.** We used CTCF and RNAPII loops identified in the ChIA-PET experiment [6]. Two types of CTCF loops were analysed separately: (1) loops with a CTCF motive in convergent orientation (64.5%; 22,709 loops) and (2) loops with a CTCF motive oriented in the same direction, i.e., tandem loops (33.1%; 11,674 loops). All RNAPII loops were analysed together (71,371 loops). We investigated the importance of chromatin marks in compartment segregation in each of these loop types separately and in genomic windows of 100 kb for comparison. We did not consider loops that were partially covered by both compartments (see Supplementary Information). For the feature selection and classifier evaluation we used 70.9% loops out of all the CTCF convergent loops (79.1% and 99.9% for CTCF tandem and RNAPII loops respectively). Out of the CTCF convergent loops, 62.7% were in compartment A and the rest were in compartment B (72.9%, 80.4% and 37.3% of CTCF tandem, RNAPII loops and genomic windows, respectively, were in compartment A).

**Genomic marks.** We collected a range of genomic data that characterize chromatin state and chromatin binding proteins in GM12878 cells.

ENCODE project data [10]:

1. DNA methylation at CpG sites Methyl Array data (GEO Accession: GSM999376), Methyl Reduced Representation Bisulfite Sequencing (RRBS) (GSM683906, GSM683927);
2. ChIP-seq data for chemical modifications (methylation, acetylation) to the histone proteins, particularly: H3K4me3, H3K27ac, H3K9ac, H4K20me1, H3K4me1, H3K4me2, H3K79me2, H3K36me3, H3K27me3, H3K9me3 and H2AZ (histone alternative variant);
3. ChIP-seq data for 87 DNA binding proteins;
4. Open chromatin data: DNase-seq, FAIRE-seq, ENCODE synthesis. This is ENCODE synthesis of evidence from different assays: DNase I hypersensitivity (HS), Formaldehyde-Assisted Isolation of Regulatory Elements (FAIRE), and chromatin immunoprecipitation (ChIP) for select regulatory factors (PolII, CTCF, c-Myc). This indicates the regions of the DNA available for direct interaction with non-histone proteins and RNA;
5. Genome segmentation: ChromHMM [28], Segway [29], ENCODE synthesis [30], the old ChromHMM method based only on histone modifications [31]. Using two different unsupervised machine learning techniques (ChromHMM and Segway), the genome was automatically segmented into disjoint segments. Each segment belongs to one of a few specific genomic “states” which is assigned an intuitive label (“active” for promoters (also inactive promoters), enhancers, transcription-associated, insulators, “repressed” for Polycomb repressed, “heterochromatin” for heterochromatin, repetitive, copy number variation regions). These methods used ENCODE ChIP-seq, DNase-seq, and FAIRE-seq data. Regions that were not assigned to “heterochromatin” not “repressed” classes by the segmentation methods went to the “active” class;
6. RNA-seq transcription data.

High-throughput experiments aside from the ENCODE project:

1. ATAC-seq [32] data. Assay for transposase-accessible chromatin using sequencing is an alternative method to detect open chromatin;
2. GRO-seq [33], Bru-seq data [34]. These are experiments that aim to identify transcription start sites from nascent RNA;
3. Repli-seq (GSE51334) [35]. Higher replication time scores correspond to earlier replication;
4. GC percentage in the genome.

Each loop in the dataset was assigned a single value for each feature. Features were genomic data in varied formats: the fraction of the loop covered by the peak signal (“fraction”), the sum or the mean of peak signal along the loop (“mean”, “sum”), the mean of the coverage signal (“whole signal mean”). Peak information was taken from the bed format files and coverage signal from bigwig files. The links to all the datasets are in Supplementary Information.

**Compartment classification.** Once the features of a particular type of loop or genomic window were computed, their association with compartment A or B was investigated as a pattern classification problem. This may be defined as a function f: X→C, where X denotes an instance in 428 dimensions and C denotes its class label. Conventionally, instances are randomly divided into two sets, one for training and the other for prediction with the trained model. However, in such an approach, prediction performance varies in different runs, and consequently classification power of the features is not fully leveraged. On the contrary, the cross-validation approach partitions the instances into p segments that are considered as the test set one at a time while the remaining p-1 segments are considered together as the training set. The classifier is trained and used for prediction p times and the mean performance is considered for assessment.

As different classifiers are good for different types of samples, we did not rely on a single classification model. Rather, seven leading classifiers viz. Naïve Bayes, K-Nearest Neighbors, Linear Discriminant Analysis, AdaBoost, Support Vector Machine, Multi-layer Perceptron and Random Forest were employed to examine the classification power of the computed features. In the Naive Bayes classifier, Gaussian distribution is used to estimate the likelihood of a sample in Compartment A or B, and hence it is referred to as Gaussian Naive Bayes, or GNB. In the KNN classifier, the 5 neighbours nearest to a recall sample are considered and the majority of their class labels give the predicted class. Radial basis function is used as the kernel of the SVM classifier. A three-layer architecture is employed in the MLP classifier, where the input layer consists of 428 dummy neurons, the hidden layer consists of 100 neurons with ReLU activation function and the output layer consists of 2 neurons with softmax activation function. Outputs of every neuron are passed to the inputs of the neurons of the next layer. Only 150 trees are built in the RFO classifier. Other parameters are the default as per the scikit-learn Python package.

**Feature ranking.** Upon observation of the fact that the features can classify genomic loops or windows into compartment A or B very well, we investigated the role of different genomic marks in the compartmentalization phenomena. To do so, two prospective feature fitness measures were employed in this work. The first one is the relative importance (RI) of the feature values, an important statistical measure from the Monte Carlo Feature Selection (MCFS) method based on decision trees [9,11], and the second one is the ANOVA f-statistic that comes from the statistical ANOVA test. The set of parameters applied to MCFS is available in Supplementary Information. The MCFS RI, ANOVA f-statistics and Product values are normalize to be between 0 and 1 when visualized on the figures.

Primarily, ranking of the features is performed on the basis of the product of RI and ANOVA f-statistic. In parallel, individual feature performance in classifying compartments is evaluated by conducting a 10-fold stratified cross-validation experiment with the RFO classifier. It may be noted that we chose the RFO classifier for this task since it was found to be the best performer in terms of AUC in most of our experiments. Keeping the feature rank and classification performance in view, genomic marks corresponding to high-performing features were identified and analysed.

## 5. Conclusions

The best simple indicator of compartmentalization is DNase-seq open chromatin measurement for CTCF loops, H3K4me1 for RNAPII loops, and H3K79me2 for genomic windows. The most valuable representation of these features for loops is by calculation of the fraction of the loop that is covered by ChIP-seq peak and for genomic windows by calculation of the mean of the whole signal. Chromatin state prediction methods that indicate active elements like promoters, enhancers or heterochromatin enhance the prediction of loop segregation into compartments above what epigenetic modifications alone can indicate. RUNX3 transcription factor best indicates compartments among DNA binding proteins in loops and RNAPII in genomic windows covering all the genome. In contrast, H3K9me3, H4K20me1, H3K27me3 histone modifications and GC content poorly indicate compartments. The ranking of the genomic marks presented here is a useful guide for the further studies on the factors that are responsible for compartmentalization. It is also a helpful hint on chromatin modification that can be used as compartment indicator in microscopic studies.

## Figures and Tables

**Table 1:** Compartment prediction performance using various genomic marks of CTCF convergent loops for various classifiers (all 428 features are considered for classification).

Classifier	R	P	F-Score	ACC	AUC	SD	RMSE
GNB	0.8139	0.8510	0.8179	0.8139	0.9138	0.0301	0.4296
KNN	0.8876	0.8912	0.8881	0.8876	0.9357	0.0234	0.2924
LDA	0.9175	0.9185	0.9166	0.9175	0.9677	0.0172	0.2509
ADB	0.9142	0.9158	0.9134	0.9142	0.9703	0.0123	0.4697
SVM	0.9264	0.9276	0.9253	0.9264	0.9689	0.0218	0.2353
MLP	0.9253	0.9262	0.9249	0.9253	0.9728	0.0167	0.2515
RFO	0.9209	0.9231	0.9195	0.9209	0.9732	0.0147	0.2419
**Mean**	**0.9008**	**0.9076**	**0.9008**	**0.9008**	**0.9575**	**0.0195**	**0.3102**

**Table 2:** The highest position in the ranking of different genomic marks for loops and genomic windows. All DNA binding proteins are represented together here.

The Highest Place in the Ranking				
Assay Type	CTCF Convergent	CTCF Tandem	RNAPII	100 kb Windows
Genome segmentation	1	1	1	1
Open chromatin	7	7	19	13
H3K4me1	10	9	7	8
H3K4me2	11	10	10	9
H3K9ac	13	13	17	11
DNA binding protein	14	19	35	42
H3K27ac	15	17	14	25
RNA-seq	18	16	9	36
H3K4me3	19	14	16	45
H3K36me3	23	24	12	12
H3K79me2	25	20	13	6
Nascent RNA	26	28	14	32
Replication time	30	34	120	43
H2A.Z	54	55	33	37
DNA methylation at CpG sites	60	53	70	151
H3K27me3	104	56	30	345
H4K20me1	119	102	45	194
H3K9me3	336	327	203	270
GC%	409	384	51	84

**Table 3:** Compartment prediction performance using various genomic marks of CTCF tandem loops for various classifiers (all 428 features are considered for classification).

Classifier	R	P	F-Score	ACC	AUC	SD	RMSE
GNB	0.7122	0.8259	0.7318	0.7122	0.8665	0.0331	0.5354
KNN	0.8613	0.8648	0.8622	0.8613	0.8942	0.0292	0.3226
LDA	0.8912	0.8897	0.8887	0.8912	0.9365	0.0246	0.2846
ADB	0.8946	0.8942	0.8927	0.8946	0.9409	0.0201	0.4778
SVM	0.8976	0.8962	0.8938	0.8976	0.9287	0.036	0.2736
MLP	0.8930	0.8928	0.8919	0.893	0.9379	0.0216	0.3038
RFO	0.9004	0.8997	0.8962	0.9004	0.9411	0.0235	0.2736
**Mean**	**0.8643**	**0.8805**	**0.8653**	**0.8643**	**0.9208**	**0.0269**	**0.3531**

**Table 4:** Compartment prediction performance using various genomic marks of RNA Pol II loops for various classifiers (all 428 features are considered for classification).

Classifier	R	P	F-Score	ACC	AUC	SD	RMSE
GNB	0.4544	0.7952	0.4963	0.4544	0.7736	0.0692	0.7372
KNN	0.8744	0.8639	0.8629	0.8744	0.7944	0.0648	0.3253
LDA	0.8801	0.8735	0.8713	0.8801	0.8657	0.0567	0.3108
ADB	0.8824	0.8754	0.8719	0.8824	0.8665	0.0563	0.4890
SVM	0.8785	0.7861	0.8243	0.8785	0.8737	0.0514	0.2730
MLP	0.8642	0.8606	0.8596	0.8642	0.8206	0.0607	0.3370
RFO	0.8910	0.8855	0.8771	0.8910	0.8676	0.0587	0.2930
**Mean**	**0.8179**	**0.8486**	**0.8091**	**0.8179**	**0.8374**	**0.0597**	**0.3950**

**Table 5:** Compartment prediction performance using various genomic marks of 100 kb genomic windows for various classifiers (all 428 genomic marks are considered for classification).

Classifier	R	P	F-Score	ACC	AUC	SD	RMSE
GNB	0.8450	0.8476	0.8424	0.8450	0.9189	0.0180	0.3930
KNN	0.8693	0.8720	0.8672	0.8693	0.9255	0.0094	0.3141
LDA	0.8983	0.9011	0.8980	0.8983	0.9655	0.0116	0.2796
ADB	0.8970	0.9009	0.8973	0.8970	0.9654	0.0107	0.4779
SVM	0.8991	0.9036	0.8992	0.8991	0.9633	0.0167	0.2703
MLP	0.8907	0.8943	0.8908	0.8907	0.9566	0.0114	0.3116
RFO	0.8960	0.8999	0.8961	0.8960	0.9645	0.0108	0.2738
**Mean**	**0.8851**	**0.8885**	**0.8844**	**0.8851**	**0.9514**	**0.0127**	**0.3315**

## Data Availability

Not applicable.

## Supplementary Materials

The following are available online at https://www.mdpi.com/article/10.3390/ijms222111591/s1, Supplementary Information, Compartment coverage and genomic features for: Table S1: CTCF convergent loops, Table S2: CTCF tandem loops, Table S3: RNAPII loops, Table S4: 100 kb genomic windows, General ranking of features for: Table S5: CTCF convergent loops, Table S6: CTCF tandem loops. Table S7: RNAPII loops, Table S8: 100 kb genomic windows.
