# Supplementary material for: Genomic Marks Associated with Chromatin Compartments in the CTCF, RNAPII Loop and Genomic Windows
Source: Int J Mol Sci. 2021 Oct 27;22(21):11591. doi: 10.3390/ijms222111591 (PMC8584073; doi:10.3390/ijms222111591)
Supplement: Supplementary file 1 [file ijms-22-11591-s001.zip › ijms-1414867-supplementary_clean.pdf]

Supplementary Information

One dimensional genomic data for the CTCF, RNAPII loops and genomic windows classification into structural compartments. 2
- Creation of the features 3
- GC Percentage 4
- DNA methylation at CpG sites 4
  - Methyl Array 4
  - Methyl Reduced Representation Bisulfite Sequencing (RRBS) 4
- Histone modification ChIP-seq 5
  - H2A.Z 6
  - H3K27ac 6
  - H3K27me3 6
  - H3K36me3 7
  - H3K4me1 7
  - H3K4me2 7
  - H3K4me3 7
  - H3K79me2 8
  - H3K9ac 8
  - H3K9me3 8
  - H4K20me1 9
- DNA binding protein ChIP-seq 9
- Open chromatin DNase-seq 16
- Open chromatin 17
  - Open chromatin FAIRE-seq 17
  - Open chromatin ENCODE synthesis 17
  - ATAC-seq 18
  - single cell ATAC-seq 18
- ENCODE genome segmentation 19
- RNA-seq 20
- Repli-seq 22
- Nascent RNA 22
  - GRO-cap 22
  - Bru-seq 23

Compartments (labels for the classification). 23

Distribution of loops of different types and genomic windows into compartment A or compartment B. 24

Parameters of MCFS 24

The data can be also visualized on three-dimensional models of GM12878 chromatin (http://3dgnome.cent.uw.edu.pl).

One dimensional genomic data for the CTCF, RNAPII loops and genomic windows classification into structural compartments.

General description of the feature ids.

Data type	Id header
H2A.Z	H001
H3K27ac	H002
H3K27me3	H003
H3K36me3	H006
H3K4me1	H009
H3K4me2	H010
H3K4me3	H011
H3K79me2	H014
H3K9ac	H015
H3K9me3	H016
H4K20me1	H017
GC Percentage	GCPe
Nascent RNA	N001-N002
Open chromatin	O001-O006
DNA methylation at CpG sites	D001-D003
RNA-seq	R001-R012
ENCODE genome segmentation	S001-S004
Replication time	P001
Transcription factor ChIP-seq	T001-T102

Creation of the features

_mean - mean of the peak values
_sum - sum of the peak values
_fraction - fraction of the loop/window covered by peaks
_mean.pbp - mean of the whole signal, without peak calling

GC Percentage

hg19.gc5Base.txt.gz - 5 bp resolution file from UCSC Genome Table
(http://genome.ucsc.edu/cgi-bin/hgTables)
Features: GCPe_mean.pbp

DNA methylation at CpG sites

Methyl Array

Description: http://genome.ucsc.edu/cgi-bin/hgTrackUi?db=hg19&g=wgEncodeHaibMethyl450

Suggested visualization parameter: score (column 5) in three categories.

1. methylated (score >= 600)
2. partially methylated (200 < score < 600)
3. unmethylated (0 < score <= 200) or NA (score = 0)

The score concern only first CpG in 50 nt regions.

Example lines:

chr16	53468112	53468162	cg00000029	577	+	53468112	53468162	128,0,128
chr3	37459206	37459256	cg00000108	913	+	37459206	37459256	255,127,0
chr3	171916037	171916087	cg00000109	870	+	171916037	171916087	255,127,0
chr1	91194674	91194724	cg00000165	123	-	91194674	91194724	0,0,205

GEO Accession: GSM999376
Machine learning [D001]: score (column 5); mean, sum, do not do fraction!
D001.methylated: score (column 5) > 600; fraction
Features: D001_mean, D001_sum, D001.methylated_fraction

Download: [D001]
http://hgdownload.cse.ucsc.edu/goldenPath/hg19/encodeDCC/wgEncodeHaibMethyl450/wgEncodeHaibMethyl450Gm12878SitesRep1.bed.gz

Methyl Reduced Representation Bisulfite Sequencing (RRBS)

“The score in this track reports the number of sequencing reads obtained for each CpG, which is often called 'coverage'. The score is capped at 1000.”

Description: http://genome.ucsc.edu/cgi-bin/hgTrackUi?db=hg19&g=wgEncodeHaibMethylRrbs

Suggested visualization threshold: score >=10

Suggested visualization parameter: percentage of methylated (column 11)
Machine learning [D002][D003]: column 11 with score (column 5) >=10; mean, sum, fraction
Features: D002_mean, D002_sum, D003_mean, D003_sum

Example lines:

track	name="SL727		MspIRRBS"		GM12878_Rep7_RRBS			MspIRRBS"		visibility=2		
	name="SL727		MspIRRBS"									
chr1	100001359		100001360		GM12878_Rep7_RRBS			1	+	100001359		
	100001360		0,255,0	1	0							
chr1	100001384		100001385		GM12878_Rep7_RRBS			1	+	100001384		
	100001385		255,0,0	1	100							
chr1	1000170	1000171	GM12878_Rep7_RRBS			52	+	1000170	1000171	105,255,0		52
	15											
chr1	1000190	1000191	GM12878_Rep7_RRBS			52	+	1000190	1000191	55,255,0	52	8

GEO Accession; Download. Data for two replicates in single bp resolution.

1. GSM683906; [D002]
   http://hgdownload.cse.ucsc.edu/goldenPath/hg19/encodeDCC/wgEncodeHaibMethylRrbs/wgEncodeHaibMethylRrbsGm12878HaibSitesRep1.bed.gz
2. GSM683927; [D003]
   http://hgdownload.cse.ucsc.edu/goldenPath/hg19/encodeDCC/wgEncodeHaibMethylRrbs/wgEncodeHaibMethylRrbsGm12878HaibSitesRep2.bed.gz

Histone modification ChIP-seq

Description of Broad Institute data:
https://genome.ucsc.edu/cgi-bin/hgTables?db=hg19&hgta_group=regulation&hgta_track=wgEncodeBroadHistone&hgta_table=wgEncodeBroadHistoneGm12878H2azStdPk&hgta_doSchema=describe+table+schema

Suggested visualization parameter: score (column 5)

Example lines of Broad Institute data:

chr22	16850291	16851951	.	643	.	6.190799	100.0	-1
chr22	16853758	16854340	.	602	.	5.696002	2.8	-1
chr22	16855214	16856146	.	587	.	5.513265	7.0	-1
chr22	16856291	16856836	.	609	.	5.778568	2.5	-1

Description of University of Washington data:
https://genome.ucsc.edu/cgi-bin/hgTables?db=hg19&hgta_group=regulation&hgta_track=wgEncodeUwHistone&hgta_table=wgEncodeUwHistoneGm12878H3k27me3StdPkRep1&hgta_doSchema=describe+table+schema

Suggested visualization parameter: signal value (column 7)

Machine learning [H001-H003, H006, H009-H011, H0014-H017]: column 7 + bigwig signal value

Example lines of University of Washington data:

chr1	713300	713450	.	0	.	91	112.768	-1	-1
chr1	713500	713650	.	0	.	25	26.7181	-1	-1
chr1	713880	714030	.	0	.	81	77.4798	-1	-1
chr1	714180	714330	.	0	.	32	106.565	-1	-1

H2A.Z

1. GSM733767 Broad Institute [H001]
   http://hgdownload.cse.ucsc.edu/goldenPath/hg19/encodeDCC/wgEncodeBroadHistone/wgEncodeBroadHistoneGm12878H2azStdPk.broadPeak.gz
   Signal file
   http://hgdownload.cse.ucsc.edu/goldenPath/hg19/encodeDCC/wgEncodeBroadHistone/wgEncodeBroadHistoneGm12878H2azStdSig.bigWig

Features: H001_mean, H001_sum, H001.bw_mean.pbp, H001_fraction

H3K27ac

1. GSM733771 Broad Institute [H002]
   http://hgdownload.cse.ucsc.edu/goldenPath/hg19/encodeDCC/wgEncodeBroadHistone/wgEncodeBroadHistoneGm12878H3k27acStdPk.broadPeak.gz
   Signal file
   http://hgdownload.cse.ucsc.edu/goldenPath/hg19/encodeDCC/wgEncodeBroadHistone/wgEncodeBroadHistoneGm12878H3k27acStdSig.bigWig

Features: H002_mean, H002_sum, H002.bw_mean.pbp, H002_fraction

H3K27me3

1. GSM733758 Broad Institute [H003]
   http://hgdownload.cse.ucsc.edu/goldenPath/hg19/encodeDCC/wgEncodeBroadHistone/wgEncodeBroadHistoneGm12878H3k27me3StdPkV2.broadPeak.gz
   Signal file
   http://hgdownload.cse.ucsc.edu/goldenPath/hg19/encodeDCC/wgEncodeBroadHistone/wgEncodeBroadHistoneGm12878H3k27me3StdSigV2.bigWig

Features: H003_mean, H003_sum, H003.bw_mean.pbp, H003_fraction

2. GSM945196 Rep1 University of Washington [H004]
   http://hgdownload.cse.ucsc.edu/goldenPath/hg19/encodeDCC/wgEncodeUwHistone/wgEncodeUwHistoneGm12878H3k27me3StdPkRep1.narrowPeak.gz

3. GSM945196 Rep2 University of Washington [H005]
http://hgdownload.cse.ucsc.edu/goldenPath/hg19/encodeDCC/wgEncodeUwHistone/wgEncodeUwHistoneGm12878H3k27me3StdPkRep2.narrowPeak.gz

H3K36me3

1. GSM733679 Broad Institute [H006]
http://hgdownload.cse.ucsc.edu/goldenPath/hg19/encodeDCC/wgEncodeBroadHistone/wgEncodeBroadHistoneGm12878H3k36me3StdPk.broadPeak.gz
Signal
http://hgdownload.cse.ucsc.edu/goldenPath/hg19/encodeDCC/wgEncodeBroadHistone/wgEncodeBroadHistoneGm12878H3k36me3StdSig.bigWig

Features: H006_mean, H006_sum, H006.bw_mean.pbp, H006_fraction

2. GSM945212 Rep1 University of Washington [H007]
http://hgdownload.cse.ucsc.edu/goldenPath/hg19/encodeDCC/wgEncodeUwHistone/wgEncodeUwHistoneGm12878H3k36me3StdPkRep1.narrowPeak.gz
3. GSM945212 Rep2 University of Washington [H008]
http://hgdownload.cse.ucsc.edu/goldenPath/hg19/encodeDCC/wgEncodeUwHistone/wgEncodeUwHistoneGm12878H3k36me3StdPkRep2.narrowPeak.gz

H3K4me1

1. GSM733772 Broad Institute [H009]
http://hgdownload.cse.ucsc.edu/goldenPath/hg19/encodeDCC/wgEncodeBroadHistone/wgEncodeBroadHistoneGm12878H3k04me1StdPkV2.broadPeak.gz
Signal
http://hgdownload.cse.ucsc.edu/goldenPath/hg19/encodeDCC/wgEncodeBroadHistone/wgEncodeBroadHistoneGm12878H3k4me1StdSig.bigWig

Features: H009_mean, H009_sum, H009.bw_mean.pbp, H009_fraction

H3K4me2

1. GSM733769 Broad Institute [H010]
http://hgdownload.cse.ucsc.edu/goldenPath/hg19/encodeDCC/wgEncodeBroadHistone/wgEncodeBroadHistoneGm12878H3k4me2StdPk.broadPeak.gz
Signal
http://hgdownload.cse.ucsc.edu/goldenPath/hg19/encodeDCC/wgEncodeBroadHistone/wgEncodeBroadHistoneGm12878H3k4me2StdSig.bigWig

Features: H010_mean, H010_sum, H010.bw_mean.pbp, H010_fraction

H3K4me3

1. GSM733708 Broad Institute [H011]
http://hgdownload.cse.ucsc.edu/goldenPath/hg19/encodeDCC/wgEncodeBroadHistone/wgEncodeBroadHistoneGm12878H3k04me3StdPkV2.broadPeak.gz

Signal
http://hgdownload.cse.ucsc.edu/goldenPath/hg19/encodeDCC/wgEncodeBroadHistone/wgEncodeBroadHistoneGm12878H3k04me3StdSigV2.bigWig
Features: H011_mean, H011_sum, H011.bw_mean.pbp, H011_fraction

2. GSM945188 University of Washington [H012]
http://hgdownload.cse.ucsc.edu/goldenPath/hg19/encodeDCC/wgEncodeUwHistone/wgEncodeUwHistoneGm12878H3k4me3StdPkRep1.narrowPeak.gz
3. GSM945188 University of Washington [H013]
http://hgdownload.cse.ucsc.edu/goldenPath/hg19/encodeDCC/wgEncodeUwHistone/wgEncodeUwHistoneGm12878H3k4me3StdPkRep2.narrowPeak.gz

H3K79me2

1. GSM733736 Broad Institute [H014]
http://hgdownload.cse.ucsc.edu/goldenPath/hg19/encodeDCC/wgEncodeBroadHistone/wgEncodeBroadHistoneGm12878H3k79me2StdPk.broadPeak.gz
Signal
http://hgdownload.cse.ucsc.edu/goldenPath/hg19/encodeDCC/wgEncodeBroadHistone/wgEncodeBroadHistoneGm12878H3k79me2StdSig.bigWig
Features: H014_mean, H014_sum, H014.bw_mean.pbp, H014_fraction

H3K9ac

1. GSM733677 Broad Institute [H015]
http://hgdownload.cse.ucsc.edu/goldenPath/hg19/encodeDCC/wgEncodeBroadHistone/wgEncodeBroadHistoneGm12878H3k9acStdPk.broadPeak.gz
Signal
http://hgdownload.cse.ucsc.edu/goldenPath/hg19/encodeDCC/wgEncodeBroadHistone/wgEncodeBroadHistoneGm12878H3k9acStdSig.bigWig
Features: H015_mean, H015_sum, H015.bw_mean.pbp, H015_fraction

H3K9me3

1. GSM733664 Broad Institute [H016]
http://hgdownload.cse.ucsc.edu/goldenPath/hg19/encodeDCC/wgEncodeBroadHistone/wgEncodeBroadHistoneGm12878H3k9me3StdPk.broadPeak.gz
Signal
http://hgdownload.cse.ucsc.edu/goldenPath/hg19/encodeDCC/wgEncodeBroadHistone/wgEncodeBroadHistoneGm12878H3k9me3StdSig.bigWig
Features: H016_mean, H016_sum, H016.bw_mean.pbp, H016_fraction

H4K20me1

1. GSM733642 Broad Institute [H017]
   http://hgdownload.cse.ucsc.edu/goldenPath/hg19/encodeDCC/wgEncodeBroadHistone/wgEncodeBroadHistoneGm12878H4k20me1StdPk.broadPeak.gz
   Signal
   http://hgdownload.cse.ucsc.edu/goldenPath/hg19/encodeDCC/wgEncodeBroadHistone/wgEncodeBroadHistoneGm12878H4k20me1StdSig.bigWig

Features: H017_mean, H017_sum, H017.bw_mean.pbp, H017_fraction

DNA binding protein ChIP-seq

ENCODE Uniform Peaks description (Processing type 1):
https://genome.ucsc.edu/cgi-bin/hgTables?db=hg19&hgta_group=regulation&hgta_track=wgEncodeAwgTfbsUniform&hgta_table=wgEncodeAwgTfbsHaibGm12878Atf2sc81188V0422111UniPk&hgta_doSchema=describe+table+schema

Example lines:

chr2	88899531	88900071	.	1000	.	556.644232292884	-1	4.7237265085109	290
chr19	42772318	42772798	.	1000	.	447.745944254712	-1	4.7237265085109	239
chr1	28974824	28975695	.	1000	.	441.812803322091	-1	4.7237265085109	365

Suggested visualization parameter: score (column 5)
Machine learning [T001]...[T102]: column 7

Features: T001_mean, T001_sum, T001_fraction … T102_mean, T102_sum, T102_fraction

Standard ChIP-seq Peaks from ENCODE/SYDH description (Processing type 2)
http://genome.ucsc.edu/cgi-bin/hgTrackUi?db=hg19&g=wgEncodeSydhTfbs

Example lines:

chrX	11810449	11811790	.	1000	.	122.14865	300.0	300.0	790
chrX	13104757	13106706	.	1000	.	46.7399	300.0	300.0	1098
chrX	18873837	18875147	.	1000	.	271.68327	300.0	300.0	559
chrX	19881040	19882430	.	1000	.	70.1822	300.0	300.0	767

Suggested visualization parameter: score (column 5)

Data with ENCODE Uniform Peak assigned:

Name UCSC_Accession Download

ATF2 wgEncodeEH002306 [T001]
http://hgdownload.cse.ucsc.edu/goldenPath/hg19/encodeDCC/wgEncodeAwgTfbsUniform/wgEncodeAwgTfbsHaibGm12878Atf2sc81188V0422111UniPk.narrowPeak.gz
ATF3 wgEncodeEH001562 [T002]
http://hgdownload.cse.ucsc.edu/goldenPath/hg19/encodeDCC/wgEncodeAwgTfbsUniform/wgEncodeAwgTfbsHaibGm12878Atf3Pcr1xUniPk.narrowPeak.gz
BATF wgEncodeEH001479 [T003]
http://hgdownload.cse.ucsc.edu/goldenPath/hg19/encodeDCC/wgEncodeAwgTfbsUniform/wgEncodeAwgTfbsHaibGm12878BatfPcr1xUniPk.narrowPeak.gz
BCL11A wgEncodeEH001486 [T004]
http://hgdownload.cse.ucsc.edu/goldenPath/hg19/encodeDCC/wgEncodeAwgTfbsUniform/wgEncodeAwgTfbsHaibGm12878Bcl11aPcr1xUniPk.narrowPeak.gz
BCL3 wgEncodeEH001658 [T005]
http://hgdownload.cse.ucsc.edu/goldenPath/hg19/encodeDCC/wgEncodeAwgTfbsUniform/wgEncodeAwgTfbsHaibGm12878Bcl3V0416101UniPk.narrowPeak.gz
BCLAF1 wgEncodeEH001563 [T006]
http://hgdownload.cse.ucsc.edu/goldenPath/hg19/encodeDCC/wgEncodeAwgTfbsUniform/wgEncodeAwgTfbsHaibGm12878Bclaf101388V0416101UniPk.narrowPeak.gz
BHLHE40 wgEncodeEH002025 [T007]
http://hgdownload.cse.ucsc.edu/goldenPath/hg19/encodeDCC/wgEncodeAwgTfbsUniform/wgEncodeAwgTfbsSydhGm12878Bhlhe40cIggmusUniPk.narrowPeak.gz
BRCA1 wgEncodeEH001830 [T008]
http://hgdownload.cse.ucsc.edu/goldenPath/hg19/encodeDCC/wgEncodeAwgTfbsUniform/wgEncodeAwgTfbsSydhGm12878Brca1a300IggmusUniPk.narrowPeak.gz
CEBPB wgEncodeEH003212 [T009]
http://hgdownload.cse.ucsc.edu/goldenPath/hg19/encodeDCC/wgEncodeAwgTfbsUniform/wgEncodeAwgTfbsHaibGm12878Cebpbsc150V0422111UniPk.narrowPeak.gz
CHD1 wgEncodeEH002823 [T010]
http://hgdownload.cse.ucsc.edu/goldenPath/hg19/encodeDCC/wgEncodeAwgTfbsUniform/wgEncodeAwgTfbsSydhGm12878Chd1a301218aIggmusUniPk.narrowPeak.gz
CHD2 wgEncodeEH001831 [T011]
http://hgdownload.cse.ucsc.edu/goldenPath/hg19/encodeDCC/wgEncodeAwgTfbsUniform/wgEncodeAwgTfbsSydhGm12878Chd2ab68301IggmusUniPk.narrowPeak.gz
CTCF wgEncodeEH000029 [T012]
denPath/hg19/encodeDCC/wgEncodeAwgTfbsUniform/wgEncodeAwgTfbsBroadGm12878CtcfUniPk.narrowPeak.gz
CTCF wgEncodeEH000394 [T013]
http://hgdownload.cse.ucsc.edu/goldenPath/hg19/encodeDCC/wgEncodeAwgTfbsUniform/wgEncodeAwgTfbsUwGm12878CtcfUniPk.narrowPeak.gz
CTCF wgEncodeEH001851 [T014]
http://hgdownload.cse.ucsc.edu/goldenPath/hg19/encodeDCC/wgEncodeAwgTfbsUniform/wgEncodeAwgTfbsSydhGm12878Ctcfsc15914c20UniPk.narrowPeak.gz
CTCF wgEncodeEH000532 [T015]
http://hgdownload.cse.ucsc.edu/goldenPath/hg19/encodeDCC/wgEncodeAwgTfbsUniform/wgEncodeAwgTfbsUtaGm12878CtcfUniPk.narrowPeak.gz
E2F4 wgEncodeEH002867 [T016]
http://hgdownload.cse.ucsc.edu/goldenPath/hg19/encodeDCC/wgEncodeAwgTfbsUniform/wgEncodeAwgTfbsSydhGm12878E2f4IggmusUniPk.narrowPeak.gz

EBF1 wgEncodeEH001832 [T017]
http://hgdownload.cse.ucsc.edu/goldenPath/hg19/encodeDCC/wgEncodeAwgTfbsUniform/wgEncodeAwgTfbsSydhGm12878Ebf1sc137065UniPk.narrowPeak.gz
EBF1 wgEncodeEH001480 [T018]
http://hgdownload.cse.ucsc.edu/goldenPath/hg19/encodeDCC/wgEncodeAwgTfbsUniform/wgEncodeAwgTfbsHaibGm12878Ebf1sc137065Pcr1xUniPk.narrowPeak.gz
EGR1 wgEncodeEH002328 [T019]
http://hgdownload.cse.ucsc.edu/goldenPath/hg19/encodeDCC/wgEncodeAwgTfbsUniform/wgEncodeAwgTfbsHaibGm12878Egr1Pcr2xUniPk.narrowPeak.gz
ELF1 wgEncodeEH001617 [T020]
http://hgdownload.cse.ucsc.edu/goldenPath/hg19/encodeDCC/wgEncodeAwgTfbsUniform/wgEncodeAwgTfbsHaibGm12878Elf1sc631V0416101UniPk.narrowPeak.gz
ELK1 wgEncodeEH002851 [T021]
http://hgdownload.cse.ucsc.edu/goldenPath/hg19/encodeDCC/wgEncodeAwgTfbsUniform/wgEncodeAwgTfbsSydhGm12878Elk112771IggmusUniPk.narrowPeak.gz
EP300 wgEncodeEH002037 [T022]
http://hgdownload.cse.ucsc.edu/goldenPath/hg19/encodeDCC/wgEncodeAwgTfbsUniform/wgEncodeAwgTfbsSydhGm12878P300bUniPk.narrowPeak.gz
EP300 wgEncodeEH002824 [T023]
http://hgdownload.cse.ucsc.edu/goldenPath/hg19/encodeDCC/wgEncodeAwgTfbsUniform/wgEncodeAwgTfbsSydhGm12878P300IggmusUniPk.narrowPeak.gz
EP300 wgEncodeEH001487 [T024]
http://hgdownload.cse.ucsc.edu/goldenPath/hg19/encodeDCC/wgEncodeAwgTfbsUniform/wgEncodeAwgTfbsHaibGm12878P300Pcr1xUniPk.narrowPeak.gz
ETS1 wgEncodeEH001564 [T025]
http://hgdownload.cse.ucsc.edu/goldenPath/hg19/encodeDCC/wgEncodeAwgTfbsUniform/wgEncodeAwgTfbsHaibGm12878Ets1Pcr1xUniPk.narrowPeak.gz
EZH2 wgEncodeEH002411 [T026]
http://hgdownload.cse.ucsc.edu/goldenPath/hg19/encodeDCC/wgEncodeAwgTfbsUniform/wgEncodeAwgTfbsBroadGm12878Ezh239875UniPk.narrowPeak.gz
FOS wgEncodeEH000622 [T027]
http://hgdownload.cse.ucsc.edu/goldenPath/hg19/encodeDCC/wgEncodeAwgTfbsUniform/wgEncodeAwgTfbsSydhGm12878CfosUniPk.narrowPeak.gz
FOXM1 wgEncodeEH002529 [T028]
http://hgdownload.cse.ucsc.edu/goldenPath/hg19/encodeDCC/wgEncodeAwgTfbsUniform/wgEncodeAwgTfbsHaibGm12878Foxm1sc502V0422111UniPk.narrowPeak.gz
GABPA wgEncodeEH001462 [T029]
http://hgdownload.cse.ucsc.edu/goldenPath/hg19/encodeDCC/wgEncodeAwgTfbsUniform/wgEncodeAwgTfbsHaibGm12878GabpPcr2xUniPk.narrowPeak.gz
IKZF1 wgEncodeEH002811 [T030]
http://hgdownload.cse.ucsc.edu/goldenPath/hg19/encodeDCC/wgEncodeAwgTfbsUniform/wgEncodeAwgTfbsSydhGm12878Ikzf1iknuclaUniPk.narrowPeak.gz
IRF4 wgEncodeEH001484 [T031]
http://hgdownload.cse.ucsc.edu/goldenPath/hg19/encodeDCC/wgEncodeAwgTfbsUniform/wgEncodeAwgTfbsHaibGm12878Irf4sc6059Pcr1xUniPk.narrowPeak.gz
JUND wgEncodeEH000639 [T032]
http://hgdownload.cse.ucsc.edu/goldenPath/hg19/encodeDCC/wgEncodeAwgTfbsUniform/wgEncodeAwgTfbsSydhGm12878JundUniPk.narrowPeak.gz

MAX wgEncodeEH002806 [T033]
http://hgdownload.cse.ucsc.edu/goldenPath/hg19/encodeDCC/wgEncodeAwgTfbsUniform/wgEncodeAwgTfbsSydhGm12878MaxIggmusUniPk.narrowPeak.gz
MAZ wgEncodeEH002852 [T034]
http://hgdownload.cse.ucsc.edu/goldenPath/hg19/encodeDCC/wgEncodeAwgTfbsUniform/wgEncodeAwgTfbsSydhGm12878Mazab85725IggmusUniPk.narrowPeak.gz
MEF2A wgEncodeEH001565 [T035]
http://hgdownload.cse.ucsc.edu/goldenPath/hg19/encodeDCC/wgEncodeAwgTfbsUniform/wgEncodeAwgTfbsHaibGm12878Mef2aPcr1xUniPk.narrowPeak.gz
MEF2C wgEncodeEH001648 [T036]
http://hgdownload.cse.ucsc.edu/goldenPath/hg19/encodeDCC/wgEncodeAwgTfbsUniform/wgEncodeAwgTfbsHaibGm12878Mef2csc13268V0416101UniPk.narrowPeak.gz
MTA3 wgEncodeEH002329 [T037]
http://hgdownload.cse.ucsc.edu/goldenPath/hg19/encodeDCC/wgEncodeAwgTfbsUniform/wgEncodeAwgTfbsHaibGm12878Mta3sc81325V0422111UniPk.narrowPeak.gz
MXI1 wgEncodeEH002026 [T038]
http://hgdownload.cse.ucsc.edu/goldenPath/hg19/encodeDCC/wgEncodeAwgTfbsUniform/wgEncodeAwgTfbsSydhGm12878Mxi1IggmusUniPk.narrowPeak.gz
MYC wgEncodeEH000547 [T039]
http://hgdownload.cse.ucsc.edu/goldenPath/hg19/encodeDCC/wgEncodeAwgTfbsUniform/wgEncodeAwgTfbsUtaGm12878CmycUniPk.narrowPeak.gz
NFATC1 wgEncodeEH002307 [T040]
http://hgdownload.cse.ucsc.edu/goldenPath/hg19/encodeDCC/wgEncodeAwgTfbsUniform/wgEncodeAwgTfbsHaibGm12878Nfatc1sc17834V0422111UniPk.narrowPeak.gz
NFE2 wgEncodeEH001808 [T041]
http://hgdownload.cse.ucsc.edu/goldenPath/hg19/encodeDCC/wgEncodeAwgTfbsUniform/wgEncodeAwgTfbsSydhGm12878Nfe2sc22827UniPk.narrowPeak.gz
NFIC wgEncodeEH002343 [T042]
http://hgdownload.cse.ucsc.edu/goldenPath/hg19/encodeDCC/wgEncodeAwgTfbsUniform/wgEncodeAwgTfbsHaibGm12878Nficsc81335V0422111UniPk.narrowPeak.gz
NFYA wgEncodeEH002064 [T043]
http://hgdownload.cse.ucsc.edu/goldenPath/hg19/encodeDCC/wgEncodeAwgTfbsUniform/wgEncodeAwgTfbsSydhGm12878NfyaIggmusUniPk.narrowPeak.gz
NFYB wgEncodeEH002065 [T044]
http://hgdownload.cse.ucsc.edu/goldenPath/hg19/encodeDCC/wgEncodeAwgTfbsUniform/wgEncodeAwgTfbsSydhGm12878NfybIggmusUniPk.narrowPeak.gz
NR2C2 wgEncodeEH000697 [T045]
http://hgdownload.cse.ucsc.edu/goldenPath/hg19/encodeDCC/wgEncodeAwgTfbsUniform/wgEncodeAwgTfbsSydhGm12878Tr4UniPk.narrowPeak.gz
NRF1 wgEncodeEH001846 [T046]
http://hgdownload.cse.ucsc.edu/goldenPath/hg19/encodeDCC/wgEncodeAwgTfbsUniform/wgEncodeAwgTfbsSydhGm12878Nrf1IggmusUniPk.narrowPeak.gz
PAX5 wgEncodeEH001489 [T047]
http://hgdownload.cse.ucsc.edu/goldenPath/hg19/encodeDCC/wgEncodeAwgTfbsUniform/wgEncodeAwgTfbsHaibGm12878Pax5c20Pcr1xUniPk.narrowPeak.gz
PAX5 wgEncodeEH001495 [T048]
http://hgdownload.cse.ucsc.edu/goldenPath/hg19/encodeDCC/wgEncodeAwgTfbsUniform/wgEncodeAwgTfbsHaibGm12878Pax5n19Pcr1xUniPk.narrowPeak.gz

PBX3 wgEncodeEH001477 [T049]
http://hgdownload.cse.ucsc.edu/goldenPath/hg19/encodeDCC/wgEncodeAwgTfbsUniform/wgEncodeAwgTfbsHaibGm12878Pbx3Pcr1xUniPk.narrowPeak.gz
PML wgEncodeEH002308 [T050]
http://hgdownload.cse.ucsc.edu/goldenPath/hg19/encodeDCC/wgEncodeAwgTfbsUniform/wgEncodeAwgTfbsHaibGm12878Pmlsc71910V0422111UniPk.narrowPeak.gz
POLR2A wgEncodeEH000626 [T051]
http://hgdownload.cse.ucsc.edu/goldenPath/hg19/encodeDCC/wgEncodeAwgTfbsUniform/wgEncodeAwgTfbsSydhGm12878Pol2UniPk.narrowPeak.gz
POLR2A wgEncodeEH000708 [T052]
http://hgdownload.cse.ucsc.edu/goldenPath/hg19/encodeDCC/wgEncodeAwgTfbsUniform/wgEncodeAwgTfbsSydhGm12878Pol2IggmusUniPk.narrowPeak.gz
POLR2A wgEncodeEH001858 [T053]
http://hgdownload.cse.ucsc.edu/goldenPath/hg19/encodeDCC/wgEncodeAwgTfbsUniform/wgEncodeAwgTfbsSydhGm12878Pol2s2IggmusUniPk.narrowPeak.gz
POLR2A wgEncodeEH000592 [T054]
http://hgdownload.cse.ucsc.edu/goldenPath/hg19/encodeDCC/wgEncodeAwgTfbsUniform/wgEncodeAwgTfbsUtaGm12878Pol2UniPk.narrowPeak.gz
POLR2A wgEncodeEH001463 [T055]
http://hgdownload.cse.ucsc.edu/goldenPath/hg19/encodeDCC/wgEncodeAwgTfbsUniform/wgEncodeAwgTfbsHaibGm12878Pol2Pcr2xUniPk.narrowPeak.gz
POLR2A wgEncodeEH001517 [T056]
http://hgdownload.cse.ucsc.edu/goldenPath/hg19/encodeDCC/wgEncodeAwgTfbsUniform/wgEncodeAwgTfbsHaibGm12878Pol24h8Pcr1xUniPk.narrowPeak.gz
POLR3G wgEncodeEH000645 [T057]
http://hgdownload.cse.ucsc.edu/goldenPath/hg19/encodeDCC/wgEncodeAwgTfbsUniform/wgEncodeAwgTfbsSydhGm12878Pol3UniPk.narrowPeak.gz
POU2F2 wgEncodeEH001475 [T058]
http://hgdownload.cse.ucsc.edu/goldenPath/hg19/encodeDCC/wgEncodeAwgTfbsUniform/wgEncodeAwgTfbsHaibGm12878Pou2f2Pcr1xUniPk.narrowPeak.gz
RAD21wgEncodeEH000749 [T059]
http://hgdownload.cse.ucsc.edu/goldenPath/hg19/encodeDCC/wgEncodeAwgTfbsUniform/wgEncodeAwgTfbsSydhGm12878Rad21IggrabUniPk.narrowPeak.gz
RAD21wgEncodeEH001640 [T060]
http://hgdownload.cse.ucsc.edu/goldenPath/hg19/encodeDCC/wgEncodeAwgTfbsUniform/wgEncodeAwgTfbsHaibGm12878Rad21V0416101UniPk.narrowPeak.gz
RCOR1 wgEncodeEH002841 [T061]
http://hgdownload.cse.ucsc.edu/goldenPath/hg19/encodeDCC/wgEncodeAwgTfbsUniform/wgEncodeAwgTfbsSydhGm12878Corestsc30189IggmusUniPk.narrowPeak.gz
RELA wgEncodeEH000690 [T062]
http://hgdownload.cse.ucsc.edu/goldenPath/hg19/encodeDCC/wgEncodeAwgTfbsUniform/wgEncodeAwgTfbsSydhGm12878NfkbTnfaIggrabUniPk.narrowPeak.gz
REST wgEncodeEH002314 [T063]
http://hgdownload.cse.ucsc.edu/goldenPath/hg19/encodeDCC/wgEncodeAwgTfbsUniform/wgEncodeAwgTfbsHaibGm12878NrsfPcr1xUniPk.narrowPeak.gz
RFX5 wgEncodeEH001810 [T064]
http://hgdownload.cse.ucsc.edu/goldenPath/hg19/encodeDCC/wgEncodeAwgTfbsUniform/wgEncodeAwgTfbsSydhGm12878Rfx5200401194IggmusUniPk.narrowPeak.gz

RUNX3 wgEncodeEH002330 [T065]
http://hgdownload.cse.ucsc.edu/goldenPath/hg19/encodeDCC/wgEncodeAwgTfbsUniform/wgEncodeAwgTfbsHaibGm12878Runx3sc101553V0422111UniPk.narrowPeak.gz
RXRA wgEncodeEH001541 [T066]
http://hgdownload.cse.ucsc.edu/goldenPath/hg19/encodeDCC/wgEncodeAwgTfbsUniform/wgEncodeAwgTfbsHaibGm12878RxraPcr1xUniPk.narrowPeak.gz
SIN3A wgEncodeEH002868 [T067]
http://hgdownload.cse.ucsc.edu/goldenPath/hg19/encodeDCC/wgEncodeAwgTfbsUniform/wgEncodeAwgTfbsSydhGm12878Sin3anb6001263IggmusUniPk.narrowPeak.gz
SIX5 wgEncodeEH001542 [T068]
http://hgdownload.cse.ucsc.edu/goldenPath/hg19/encodeDCC/wgEncodeAwgTfbsUniform/wgEncodeAwgTfbsHaibGm12878Six5Pcr1xUniPk.narrowPeak.gz
SMC3 wgEncodeEH001833 [T069]
http://hgdownload.cse.ucsc.edu/goldenPath/hg19/encodeDCC/wgEncodeAwgTfbsUniform/wgEncodeAwgTfbsSydhGm12878Smc3ab9263IggmusUniPk.narrowPeak.gz
SP1 wgEncodeEH001496 [T070]
http://hgdownload.cse.ucsc.edu/goldenPath/hg19/encodeDCC/wgEncodeAwgTfbsUniform/wgEncodeAwgTfbsHaibGm12878Sp1Pcr1xUniPk.narrowPeak.gz
SPI1 wgEncodeEH001476 [T071]
http://hgdownload.cse.ucsc.edu/goldenPath/hg19/encodeDCC/wgEncodeAwgTfbsUniform/wgEncodeAwgTfbsHaibGm12878Pu1Pcr1xUniPk.narrowPeak.gz
SRF wgEncodeEH001464 [T072]
http://hgdownload.cse.ucsc.edu/goldenPath/hg19/encodeDCC/wgEncodeAwgTfbsUniform/wgEncodeAwgTfbsHaibGm12878SrfPcr2xUniPk.narrowPeak.gz
STAT1 wgEncodeEH001852 [T073]
http://hgdownload.cse.ucsc.edu/goldenPath/hg19/encodeDCC/wgEncodeAwgTfbsUniform/wgEncodeAwgTfbsSydhGm12878Stat1UniPk.narrowPeak.gz
STAT3 wgEncodeEH001811 [T074]
http://hgdownload.cse.ucsc.edu/goldenPath/hg19/encodeDCC/wgEncodeAwgTfbsUniform/wgEncodeAwgTfbsHaibGm12878Stat5asc74442V0422111UniPk.narrowPeak.gz
STAT5A wgEncodeEH002321 [T075]
http://hgdownload.cse.ucsc.edu/goldenPath/hg19/encodeDCC/wgEncodeAwgTfbsUniform/wgEncodeAwgTfbsHaibGm12878Stat5asc74442V0422111UniPk.narrowPeak.gz
TAF1 wgEncodeEH001478 [T076]
http://hgdownload.cse.ucsc.edu/goldenPath/hg19/encodeDCC/wgEncodeAwgTfbsUniform/wgEncodeAwgTfbsHaibGm12878Taf1Pcr1xUniPk.narrowPeak.gz
TBL1XR1 wgEncodeEH002853 [T077]
http://hgdownload.cse.ucsc.edu/goldenPath/hg19/encodeDCC/wgEncodeAwgTfbsUniform/wgEncodeAwgTfbsSydhGm12878Tblr1ab24550IggmusUniPk.narrowPeak.gz
TBP wgEncodeEH001798 [T078]
http://hgdownload.cse.ucsc.edu/goldenPath/hg19/encodeDCC/wgEncodeAwgTfbsUniform/wgEncodeAwgTfbsSydhGm12878TbpIggmusUniPk.narrowPeak.gz
TCF12 wgEncodeEH001485 [T079]
http://hgdownload.cse.ucsc.edu/goldenPath/hg19/encodeDCC/wgEncodeAwgTfbsUniform/wgEncodeAwgTfbsHaibGm12878Tcf12Pcr1xUniPk.narrowPeak.gz
TCF3 wgEncodeEH002315 [T080]
http://hgdownload.cse.ucsc.edu/goldenPath/hg19/encodeDCC/wgEncodeAwgTfbsUniform/wgEncodeAwgTfbsHaibGm12878Tcf3Pcr1xUniPk.narrowPeak.gz

USF1 wgEncodeEH001468 [T081]
http://hgdownload.cse.ucsc.edu/goldenPath/hg19/encodeDCC/wgEncodeAwgTfbsUniform/wgEncodeAwgTfbsHaibGm12878Usf1Pcr2xUniPk.narrowPeak.gz
USF2 wgEncodeEH001812 [T082]
http://hgdownload.cse.ucsc.edu/goldenPath/hg19/encodeDCC/wgEncodeAwgTfbsUniform/wgEncodeAwgTfbsSydhGm12878Usf2IggmusUniPk.narrowPeak.gz
WRNIP1 wgEncodeEH001787 [T083]
http://hgdownload.cse.ucsc.edu/goldenPath/hg19/encodeDCC/wgEncodeAwgTfbsUniform/wgEncodeAwgTfbsSydhGm12878WhipIggmusUniPk.narrowPeak.gz
YY1 wgEncodeEH000695 [T084]
http://hgdownload.cse.ucsc.edu/goldenPath/hg19/encodeDCC/wgEncodeAwgTfbsUniform/wgEncodeAwgTfbsSydhGm12878Yy1UniPk.narrowPeak.gz
YY1 wgEncodeEH001657 [T085]
http://hgdownload.cse.ucsc.edu/goldenPath/hg19/encodeDCC/wgEncodeAwgTfbsUniform/wgEncodeAwgTfbsHaibGm12878Yy1sc281Pcr1xUniPk.narrowPeak.gz
ZBTB33 wgEncodeEH001488 [T086]
http://hgdownload.cse.ucsc.edu/goldenPath/hg19/encodeDCC/wgEncodeAwgTfbsUniform/wgEncodeAwgTfbsHaibGm12878Zbtb33Pcr1xUniPk.narrowPeak.gz
ZEB1 wgEncodeEH001645 [T087]
http://hgdownload.cse.ucsc.edu/goldenPath/hg19/encodeDCC/wgEncodeAwgTfbsUniform/wgEncodeAwgTfbsHaibGm12878Zeb1sc25388V0416102UniPk.narrowPeak.gz
ZNF143 wgEncodeEH001853 [T088]
http://hgdownload.cse.ucsc.edu/goldenPath/hg19/encodeDCC/wgEncodeAwgTfbsUniform/wgEncodeAwgTfbsSydhGm12878Znf143166181apUniPk.narrowPeak.gz
ZNF274 wgEncodeEH001756 [T089]
http://hgdownload.cse.ucsc.edu/goldenPath/hg19/encodeDCC/wgEncodeAwgTfbsUniform/wgEncodeAwgTfbsSydhGm12878Znf274UniPk.narrowPeak.gz
ZZZ3 wgEncodeEH000698 [T090]
http://hgdownload.cse.ucsc.edu/goldenPath/hg19/encodeDCC/wgEncodeAwgTfbsUniform/wgEncodeAwgTfbsSydhGm12878Zzz3UniPk.narrowPeak.gz

Data with Standard ChIP-seq Peaks from ENCODE/SYDH assigned:

CUX1(CDP) [T091]
http://hgdownload.cse.ucsc.edu/goldenPath/hg19/encodeDCC/wgEncodeSydhTfbs/wgEncodeSydhTfbsGm12878Cdpsc6327IggmusPk.narrowPeak.gz
ESRRA [T092]
http://hgdownload.cse.ucsc.edu/goldenPath/hg19/encodeDCC/wgEncodeSydhTfbs/wgEncodeSydhTfbsGm12878ErraIggrabPk.narrowPeak.gz
FAM48A [T093]
http://hgdownload.cse.ucsc.edu/goldenPath/hg19/encodeDCC/wgEncodeSydhTfbs/wgEncodeSydhTfbsGm12878Spt20StdPk.narrowPeak.gz
IRF3 [T094]
http://hgdownload.cse.ucsc.edu/goldenPath/hg19/encodeDCC/wgEncodeSydhTfbs/wgEncodeSydhTfbsGm12878Irf3IggmusPk.narrowPeak.gz

KAT2A(GCN5) [T095]
http://hgdownload.cse.ucsc.edu/goldenPath/hg19/encodeDCC/wgEncodeSydhTfbs/wgEncodeSydhTfbsGm12878Gcn5StdPk.narrowPeak.gz
MAFK [T096]
http://hgdownload.cse.ucsc.edu/goldenPath/hg19/encodeDCC/wgEncodeSydhTfbs/wgEncodeSydhTfbsGm12878MafkIggmusPk.narrowPeak.gz
SREBP1 [T097]
http://hgdownload.cse.ucsc.edu/goldenPath/hg19/encodeDCC/wgEncodeSydhTfbs/wgEncodeSydhTfbsGm12878Srebp1IggrabPk.narrowPeak.gz
SREBP2 [T098]
e.ucsc.edu/goldenPath/hg19/encodeDCC/wgEncodeSydhTfbs/wgEncodeSydhTfbsGm12878Srebp2IggrabPk.narrowPeak.gz
ZNF274 [T099]
http://hgdownload.cse.ucsc.edu/goldenPath/hg19/encodeDCC/wgEncodeAwgTfbsUniform/wgEncodeAwgTfbsSydhGm12878Znf274UniPk.narrowPeak.gz
ZNF384 [T100]
http://hgdownload.cse.ucsc.edu/goldenPath/hg19/encodeDCC/wgEncodeSydhTfbs/wgEncodeSydhTfbsGm12878Znf384hpa004051IggmusPk.narrowPeak.gz

Data without combined replicates:
CREB1 (Rep 1) [T101]
http://hgdownload.cse.ucsc.edu/goldenPath/hg19/encodeDCC/wgEncodeHaibTfbs/wgEncodeHaibTfbsGm12878Creb1sc240V0422111PkRep1.broadPeak.gz
CREB1 (Rep 2) [T102]
http://hgdownload.cse.ucsc.edu/goldenPath/hg19/encodeDCC/wgEncodeHaibTfbs/wgEncodeHaibTfbsGm12878Creb1sc240V0422111PkRep2.broadPeak.gz

Open chromatin DNase-seq

Data description:
https://genome.ucsc.edu/cgi-bin/hgTrackUi?hgsid=561795937_jVEsl5EEwrXniKOQX5ead2GZ1Aj3&c=chr7&g=wgEncodeDNAseSuper

DNase Hypersensitivity data description:
http://genome.ucsc.edu/cgi-bin/hgTrackUi?db=hg19&g=wgEncodeAwgDnaseUniform

Machine learning [O001, O002]: column 7
[O002, O006] - bigwig signal x2
Download: [O001]
http://hgdownload.cse.ucsc.edu/goldenPath/hg19/encodeDCC/wgEncodeAwgDnaseUniform/wgEncodeAwgDnaseUwdukeGm12878UniPk.narrowPeak.gz

Features: O001_mean, O001_sum, O001_fraction

Download: [O006] - peaks from different analysis pipeline
http://hgdownload.cse.ucsc.edu/goldenPath/hg19/encodeDCC/wgEncodeOpenChromDnase/wgEncodeOpenChromDnaseGm12878Pk.narrowPeak.gz
Signal 1 - [O006s1]
http://hgdownload.cse.ucsc.edu/goldenPath/hg19/encodeDCC/wgEncodeOpenChromDnase/wgEncodeOpenChromDnaseGm12878Sig.bigWig
Signal 2 - [O006s2]
http://hgdownload.cse.ucsc.edu/goldenPath/hg19/encodeDCC/wgEncodeOpenChromDnase/wgEncodeOpenChromDnaseGm12878BaseOverlapSignal.bigWig

Features: O006_mean, O006_sum, O006_fraction, O006s1.bw_mean.pbp, O006s2.bw_mean.pbp

Open chromatin

Open chromatin FAIRE-seq

FAIRE-seq data description https://genome.ucsc.edu/cgi-bin/hgTrackUi?hgsid=561779245_gadHNsG3pzt6A3OwqrWCOYobw32l&c=chr7&g=wgEncodeOpenChromFaire

Download: [O002]
http://hgdownload.cse.ucsc.edu/goldenPath/hg19/encodeDCC/wgEncodeOpenChromFaire/wgEncodeOpenChromFaireGm12878Pk.narrowPeak.gz
Signal 1 [O002s1]
http://hgdownload.cse.ucsc.edu/goldenPath/hg19/encodeDCC/wgEncodeOpenChromFaire/wgEncodeOpenChromFaireGm12878Sig.bigWig
Signal 2 [O002s2]
http://hgdownload.cse.ucsc.edu/goldenPath/hg19/encodeDCC/wgEncodeOpenChromFaire/wgEncodeOpenChromFaireGm12878BaseOverlapSignal.bigWig

Features: O002_mean, O002_sum, O002_fraction, O002s1.bw_mean.pbp, O002s2.bw_mean.pbp

Open chromatin ENCODE synthesis

ENCODE synthesis of evidence from different assays: DNaseI hypersensitivity (HS), Formaldehyde-Assisted Isolation of Regulatory Elements (FAIRE), and chromatin immunoprecipitation (ChIP) for select regulatory factors (PolII, CTCF, c-Myc).

Data description: https://genome.ucsc.edu/cgi-bin/hgTrackUi?db=hg19&g=wgEncodeOpenChromSynth

Example lines:

```
chr1	10166	10376	FAIREOnly_1	1000	.	10166	10376	153,0,0	0.99	0.00160	0.00
	0.01580	1.68	0.02650	1.51	0.00840	0.00	0.03360	0.00	3
```

chr1	91363	91505	ChIPOnly_1	1000	.	91363	91505	255,0,255	0.24	0.01320	0.62	0.00320	0.00	0.00000	0.00	0.22470	3.79	0.01890	0.00	4
chr1	237659	237873	ChIPOnly_2	1000	.	237659	237873	255,0,255	0	0.00570	0.00	0.00220	0.00	0.00150	0.00	0.21420	3.62	0.00000	0.00	4
chr1	521439	521613	ChIPOnly_3	1000	.	521439	521613	255,0,255	0.52	0.02080	1.05	0.00300	0.00	0.00120	0.00	0.45450	7.33	0.00000	0.00	4
chr1	545991	546181	ChIPOnly_4	1000	.	545991	546181	255,0,255	0	0.00110	0.00	0.00000	0.00	0.00130	0.00	0.17650	3.03	0.01410	0.00	4
chr1	713841	714424	Valid_1	1000	.	713841	714424	0,0,0	11.17	0.17830	11.00	0.01560	1.65	0.14410	9.54	0.13180	2.33	0.05530	2.18	1

Suggested visualization threshold and parameter: only three categories from column 4: Valid, OpenChrom, ChIPOnly

Machine learning [O003]:1 for all regions (binary)
Features: O003_sum, O003_fraction

Download: [O003]
http://hgdownload.cse.ucsc.edu/goldenPath/hg19/encodeDCC/wgEncodeOpenChromSynth/wgEncodeOpenChromSynthGm12878Pk.bed.gz

ATAC-seq

Data are not from ENCODE
Data description:
https://www.ncbi.nlm.nih.gov/geo/query/acc.cgi?acc=GSE47753

“Peak files are in UCSC broadPeak format, which includes the following columns: chromosome, start, stop, name, arbitrary score (1000), strand, read count within peaks, -log10(1-posterior probability), -log10(qvalue).”

Suggested visualization parameter: read count within peaks (column 7)

Example lines:

chr1	9976	10725	id.1	1000	+	212	15.95458977019100	16.00000000000000
chr1	235351	235950	id.2	1000	+	78	3.74332637619407	4.95770682345444
chr1	564976	570525	id.3	1000	+	2985	15.95458977019100	16.00000000000000
chr1	713701	714750	id.4	1000	+	268	15.95458977019100	16.00000000000000

1. GSE47753 [O004]
   http://www.ncbi.nlm.nih.gov/geo/download/?acc=GSE47753&format=file&file=GSE47753%5FGM12878%5FATACseq%5F50k%5FAllReps%5FZINBA%5Fpp08%2Ebed%2Egz

Machine learning [O004]: read count within peaks (column 7)
Features: O004_mean, O004_sum, O004_fraction

single cell ATAC-seq

PMID: 26083756

1. GSE65360 [O005]
   http://www.ncbi.nlm.nih.gov/geo/download/?acc=GSE65360&format=file&file=GSE65360%5Fsingle%2DGM12878%2Epeaks%2Ebed%2Egz

Suggested visualization parameter: None
Machine learning [O005]:1 for all regions (binary)
Features: O005_sum, O005_fraction

Example lines:

chr1	713880	714380
chr1	762641	763141
chr1	805000	805500

ENCODE genome segmentation

Discrete annotation maps of regulatory regions and other chromatin elements.
Description:
http://genome.ucsc.edu/cgi-bin/hgTrackUi?db=hg19&g=wgEncodeAwgSegmentation

PMID: 23221638

Example lines:

chr1	10000	10271	CTCF	1000	.	10000	10271	10,190,254
chr1	10400	10605	T	1000	.	10400	10605	0,176,80
chr1	10605	15800	R	1000	.	10605	15800	127,127,127
chr1	15800	18820	T	1000	.	15800	18820	0,176,80
chr1	18820	109711	R	1000	.	18820	109711	127,127,127

Suggested visualization parameter: segment category (column 4)
Machine learning [S001-S003]:1 for all regions (binary) in the category
S001 categories:

Active - 1 for all regions but repressed

1. Combined Download [S001]:
   http://hgdownload.cse.ucsc.edu/goldenPath/hg19/encodeDCC/wgEncodeAwgSegmentation/wgEncodeAwgSegmentationCombinedGm12878.bed.gz

Features: S001.active_fraction, S001.repressed_fraction

2. ChromHMM [S002]:
   http://hgdownload.cse.ucsc.edu/goldenPath/hg19/encodeDCC/wgEncodeAwgSegmentation/wgEncodeAwgSegmentationChromhmmGm12878.bed.gz

Features: S002.active_fraction, S002.heterochromatin_fraction, S002.repressed_fraction

3. Segway [S003]:
   http://hgdownload.cse.ucsc.edu/goldenPath/hg19/encodeDCC/wgEncodeAwgSegmentation/wgEncodeAwgSegmentationSegwayGm12878.bed.gz

Features: S003.active_fraction, S003.heterochromatin_fraction, S003.repressed_fraction

4. ChromHMM, only histone marks [S004]: http://hgdownload.cse.ucsc.edu/goldenPath/hg19/encodeDCC/wgEncodeBroadHmm/wgEncodeBroadHmmGm12878HMM.bed.gz

Features: S004.active_fraction, S004.heterochromatin_fraction, S004.repressed_fraction

RNA-seq

Data description:
https://genome.ucsc.edu/cgi-bin/hgTrackUi?hgsid=561843471_QiMzI3fxEUmLTlBkmKEeNP0a4NAi&c=chr7&g=wgEncodeCshlShortRnaSeq

“Contigs (continuous regions covered by uniquely alligned reads) are in BED9 format. The Contigs represent blocks of overlapping mapped reads from the pooled biological replicates. FORMAT: 9 \t seperated columns. 1-6: BED 7: Bases per Kilobase per Million mapped bases (BPKM) 8: non-parametric irreproducible discovery score (npIDR) between the replicates 9: Total mapped bases. The bedscore (column 5) is computed as min(1000,100*log(summed_bpkm*100+1)).”

Example lines:

chr1	244085	244163	contig_12	254	+	0.115	0.58	228
chr1	325159	325261	contig_18	282	+	0.148	0.30	403
chr1	462046	462240	contig_25	306	+	0.201	0.09	986

Suggested threshold: npIDR (column 8) < 0.1
Suggested visualization parameter: bed_score (column 5)
Machine learning [R001-R012]: column 7 with column 8 < 0.1

A tool to sum bigwig files https://www.ncbi.nlm.nih.gov/pmc/articles/PMC3967112/

Experiments (GEO accession; Name \t Download):

1. GSM758572; whole cell polyA- [R001] http://hgdownload.cse.ucsc.edu/goldenPath/hg19/encodeDCC/wgEncodeCshlLongRnaSeq/wgEncodeCshlLongRnaSeqGm12878CellPamContigs.bedRnaElements.gz

Features: R001_mean, R001_sum, R001_fraction

2. GSM758559; whole cell polyA+ [R002] http://hgdownload.cse.ucsc.edu/goldenPath/hg19/encodeDCC/wgEncodeCshlLongRnaSeq/wgEncodeCshlLongRnaSeqGm12878CellPapContigs.bedRnaElements.gz

Features: R002_mean, R002_sum, R002_fraction

3. GSM767852; cytosol polyA- [R003]
   http://hgdownload.cse.ucsc.edu/goldenPath/hg19/encodeDCC/wgEncodeCshlLongRnaSeq/wgEncodeCshlLongRnaSeqGm12878CytosolPamContigs.bedRnaElements.gz

Features: R003_mean, R003_sum, R003_fraction

4. GSM758560; cytosol polyA+ [R004]
   http://hgdownload.cse.ucsc.edu/goldenPath/hg19/encodeDCC/wgEncodeCshlLongRnaSeq/wgEncodeCshlLongRnaSeqGm12878CytosolPapContigs.bedRnaElements.gz

Features: R004_mean, R004_sum, R004_fraction

5. GSM984605; nucleolus total [R005]
   http://hgdownload.cse.ucsc.edu/goldenPath/hg19/encodeDCC/wgEncodeCshlLongRnaSeq/wgEncodeCshlLongRnaSeqGm12878NucleolusTotalContigs.bedRnaElements.gz

Features: R005_mean, R005_sum, R005_fraction

6. GSM767853; nucleus polyA- [R006]
   http://hgdownload.cse.ucsc.edu/goldenPath/hg19/encodeDCC/wgEncodeCshlLongRnaSeq/wgEncodeCshlLongRnaSeqGm12878NucleusPamContigs.bedRnaElements.gz

Features: R006_mean, R006_sum, R006_fraction

7. GSM765386; nucleus polyA+ [R007]
   http://hgdownload.cse.ucsc.edu/goldenPath/hg19/encodeDCC/wgEncodeCshlLongRnaSeq/wgEncodeCshlLongRnaSeqGm12878NucleusPapContigs.bedRnaElements.gz

Features: R007_mean, R007_sum, R007_fraction

8. GSM605625; TAP-only whole cell small [R008]
   http://hgdownload.cse.ucsc.edu/goldenPath/hg19/encodeDCC/wgEncodeCshlShortRnaSeq/wgEncodeCshlShortRnaSeqGm12878CellShorttotalTapContigs.bedRnaElements.gz

Features: R008_mean, R008_sum, R008_fraction

9. GSM977042; TAP-only chromatin small [R009]
   http://hgdownload.cse.ucsc.edu/goldenPath/hg19/encodeDCC/wgEncodeCshlShortRnaSeq/wgEncodeCshlShortRnaSeqGm12878ChromatinTapContigs.bedRnaElements.gz

10. GSM605627; TAP-only cytosol small [R010]
    http://hgdownload.cse.ucsc.edu/goldenPath/hg19/encodeDCC/wgEncodeCshlShortRnaSeq/wgEncodeCshlShortRnaSeqGm12878CytosolShorttotalTapContigs.bedRnaElements.gz

Features: R010_mean, R010_sum, R010_fraction

11. GSM977032; TAP-only nucleolus small [R011]
    http://hgdownload.cse.ucsc.edu/goldenPath/hg19/encodeDCC/wgEncodeCshlShortRnaSeq/wgEncodeCshlShortRnaSeqGm12878NucleolusTapContigs.bedRnaElements.gz

Features: R011_mean, R011_sum, R011_fraction

12. GSM605633; TAP-only nucleus small [R012]
    http://hgdownload.cse.ucsc.edu/goldenPath/hg19/encodeDCC/wgEncodeCshlShortRnaSeq/wgEncodeCshlShortRnaSeqGm12878NucleusShorttotalTapContigs.bedRnaElements.gz

Features: R012_mean, R012_sum, R012_fraction

Repli-seq

"Peaks - Local maxima in the wavelet-smoothed signal data corresponding to replication initiation (replication origin) zones. Higher replication time score (column 5, range 0-1000) correspond to earlier replication. Peak span is set to 1kb."

Suggested visualization parameter: Replication time score (column 5).
Machine learning [P001]: column 5, calculate mean for the loop

Example lines:

chr1	227500	228500	.	600	+	.	.	179,45,0
chr1	620500	621500	.	700	+	.	.	179,45,0
chr1	986500	987500	.	1000	+	.	.	179,45,0
chr1	1745500	1746500	.	900	+	.	.	179,45,0

1. GSE51334 [P001]
   http://hgdownload.cse.ucsc.edu/goldenPath/hg19/encodeDCC/wgEncodeUwRepliSeq/wgEncodeUwRepliSeqGm12878PkRep1.bed.gz

Features: P001_mean

Nascent RNA

GRO-cap

GRO-seq transcription start sites from nascent RNA

Suggested visualization parameter: None

Example lines:

chr1	896250	896310	M1	0	+
chr1	901850	901880	M1	0	+

1. GSE60456, processed TSS are in tss_all_gm12878.bed [N001] file in Supplementary Data Set to the publication http://www.ncbi.nlm.nih.gov/pubmed/25383968 http://www.nature.com/ng/journal/v46/n12/extref/ng.3142-S2.zip

Machine learning [N001]:1 for all regions (binary)

Features: N001_sum, N001_fraction

Bru-seq

nascent RNA sequencing

1. GSM1347660 [N002] http://www.ncbi.nlm.nih.gov/geo/download/?acc=GSM1347660&format=file&file=GSM1347660%5FGM128780h1%2Esegments%2Ebed%2Egz

“supplementary_files_format_and_content: segments.bed = extended BED format file, one record for each HMM transcription segment, where column 4 is a unique identifier for the corresponding fused and revised transcription unit in format chrom:start-end(strand), column 5 is always 0, column 7 is the segment’s HMM state (0..9), column 8 is segment RPKM, and columns 9 is the transcription unit RPKM.”

Suggested threshold: HMM state > 2 (column 7);
Suggested visualization parameter: HMM state (column 7)
Machine learning [N002]: column 8 with column 7 > 2

Features: N002_mean, N002_sum, N002_fraction

Example lines:

chr1	9500	13500	NA	0	+	9	0	NA
chr1	14500	16500	NA	0	+	8	0	NA
chr1	46500	549500	NA	0	+	0	0	NA
chr1	558500	565500	NA	0	+	3	0	NA
chr1	565500	566453	NA	0	+	7	1.81379	NA
chr1	566453	568136	chr1:566453-568136(+)	0	+	7	1.81379	3.90816
chr1	568136	569075	chr1:568136-569075(+)	0	+	7	1.81379	0.041571

Compartments (labels for the classification).

We have used compartment genomic coordinates discovered in Hi-C experiments where subcompartments were associated with 100 kb windows (Rao et al. 2014). Subcompartment A1 and A2 were associated with compartment A and compartment B1, B2, B3, B4 were associated with compartment B.

Distribution of loops of different types and genomic windows into compartment A or compartment B.

Supplementary Table. Distribution of loops of different types and genomic windows into compartment A or compartment B. Unambiguous loops/windows are those that are partially covered by both compartments.

Loop type / Window	Total number of loops / windows	Number of unambiguous loops / windows	Compartment A	Compartment B
CTCF Convergent loops	22,709	15,388	10,099	5,289
CTCF tandem loops	11,674	8,925	6,734	2,191
RNA Pol II loops	71,371	69,681	57,349	12,332
Genomic Windows	30,376	26,336	9,820	16,516

Parameters of MCFS

Parameters of MCFS applied to analysis are: projections=1000, projectionSize=0.1, splitSetSize=0, splits=10, cutoffPermutations=20,cutoffMethod="permutations", finalCV=FALSE, finalRuleset=FALSE
